# Cross‐Cultural Paediatric Neuropsychological Assessment: Key Considerations

**DOI:** 10.1002/hsr2.70959

**Published:** 2025-06-23

**Authors:** Freddie O'Donald, Clara Calia, Sanne Franzen

**Affiliations:** ^1^ Paediatric Neurodevelopmental Service, Centre for Child Health Dundee Scotland; ^2^ School of Health in Social Science University of Edinburgh Edinburgh Scotland; ^3^ Erasmus MC Department of Neurology Erasmus MC University Medical Center Rotterdam The Netherlands

**Keywords:** cross‐cultural neuropsychology, cultural adaptation, interpreter‐mediated testing, paediatric neuropsychology

## Abstract

**Background and Aims:**

Cross‐cultural paediatric neuropsychology faces important challenges in ensuring assessments are fair and valid across diverse cultural, linguistic, and socioeconomic backgrounds. This article aims to highlight key considerations and gaps in current practice, with a focus on supporting equitable neuropsychological assessment practices in increasingly diverse populations.

**Methods:**

This perspective article draws on a structured narrative review of key literature and explores core themes in the cross‐cultural neuropsychological assessment of children, including test adaptation, developmental considerations, and interpreter‐mediated assessment.

**Results:**

Three main areas are discussed: (1) the inconsistent adaptation of paediatric‐specific cognitive assessment tools across cultures, (2) the influence of cultural and educational factors on child development and test performance, and (3) the complexities of interpreter‐mediated neuropsychological assessment in paediatric settings. Although there are established tools and emerging guidance in adult neuropsychology, systematic, child‐focused cross‐cultural assessment frameworks remain underdeveloped.

**Conclusion:**

Further research is needed to develop culturally responsive, developmentally appropriate, and standardised frameworks for paediatric neuropsychological assessment. This includes improving normative data sets, refining cross‐cultural adaptation processes, and developing interpreter protocols tailored to child assessments. These efforts are essential to ensure that assessments are equitable, accurate, and clinically useful across diverse populations.

## Summary

- This perspective article outlines key challenges in cross‐cultural paediatric neuropsychology and presents essential considerations for enhancing neuropsychological assessment practices.
- By improving assessment tools, implementing structured guidelines for interpreter use, and developing culturally responsive frameworks, clinicians can enhance diagnostic accuracy and ensure fair, effective evaluations for children from diverse backgrounds.
- There remains a critical need for developing and validating cross‐culturally appropriate paediatric neuropsychological assessments.
- Given the increasing diversity of European populations, future research should prioritise the creation of inclusive, developmentally appropriate tools.
- Advancing these efforts through research and clinical practice will contribute to more equitable and culturally competent paediatric neuropsychology across Europe.

Cross‐cultural paediatric neuropsychology examines how cultural, linguistic, and socioeconomic factors influence cognitive assessments in children. As European societies become increasingly diverse, neuropsychological assessments must be adapted to account for these variations, ensuring fairness and accuracy in diagnosis and interpretation [1]. This article briefly discusses three key aspects of cross‐cultural paediatric neuropsychology: (1) the adaptation of paediatric‐specific assessment tools, (2) developmental considerations in cross‐cultural contexts, and (3) interpreter‐mediated assessments. By addressing these key areas, we highlight the need for further research to refine assessment tools, establish standardised guidelines for interpreter use, and improve diagnostic accuracy for children from diverse backgrounds. Advancing these efforts will help ensure that neuropsychological assessments are equitable, culturally fair, and clinically effective across different populations.

This perspective article is primarily aimed at clinicians, researchers, and trainees in neuropsychology working within increasingly multicultural European contexts, where the need for culturally responsive paediatric assessment tools is growing. While many of the issues discussed are globally relevant, this article focuses on broad cross‐cultural principles rather than culture‐specific comparisons, emphasising practical and developmental considerations in clinical assessment. Relevant literature was identified using a structured narrative approach focused on this article's key themes.

## 1 Cross‐Cultural Adaptation of Paediatric‐Specific Assessment Tools

Neuropsychological assessments for children often rely on standardised tools developed within specific cultural and linguistic contexts, primarily in English and the Anglo‐American context [2]. For these assessments to be effective across different populations, they require careful adaptation rather than direct translation [3]. However, adaption procedures have been inconsistently applied, leading to concerns regarding test validity [4, 5]. For example, while some studies suggest that the five‐factor structure of the Wechsler Intelligence Scale for Children, Fifth Edition (WISC‐V) is invariant across cultures [6], other research suggests methodological flaws in its adaption process. McGill et al. [5] emphasise that many translated versions of the WISC‐V lack independent validation, relying instead on score‐equating procedures that assume construct equivalence without empirical confirmation. As a result, discrepancies emerge when applying standard norms to different cultural groups. For example, Canadian children assessed using the U.S. WISC‐V norms tend to score slightly higher on non‐verbal reasoning tasks, indicating potential cross‐national differences in cognitive development and educational experiences [7]. Similar inconsistencies have been identified in international adaptions of other widely used tests, such as the NEPSY‐II, where linguistic and cultural influences impact test performance [8].

Beyond translation, cultural adaptation must involve modifications to account for educational, linguistic, and socio‐environmental influences on cognitive function. Often, adapted versions of tests may exclude certain subtests or alter item content, which may introduce bias and reduce comparability with the original version [4]. For example, the Spanish version of the WISC‐V lacks full‐scale psychometric validation and relies on equating procedures that do not adequately assess whether it measures the same constructs as the US version [9]. Such limitations highlight the need for a more rigorous framework for cross‐cultural test adaptation.

Findings from Byrd et al. [10] and Olson and Jacobson [2] further emphasise these concerns, highlighting that studies examining the impact of cultural factors on paediatric neuropsychological assessments remain scarce. Although these reviews are somewhat dated, they both highlight how adult neuropsychology has seen advances in approaching cross‐cultural assessment, but child‐focused research has lagged behind, and there remains a lack of systematic child‐specific work. It can be argued that cultural considerations in paediatric neuropsychological assessments have often been overly broad [11], and further research is needed to establish valid and reliable cross‐cultural tools that can be effectively used across diverse paediatric populations.

To address these issues, future research should prioritise developing widely applicable cross‐cultural assessments supported by normative data sets that reflect linguistic, educational, and socio‐environmental diversity. This requires standardised frameworks for cross‐cultural adaptation that extend beyond linguistic translation to account for cultural, educational, and socio‐environmental influences on cognitive performance. Establishing diverse normative data sets and ensuring that adapted assessments maintain construct validity across populations are essential steps in this process. Race‐based normative adjustments—where test scores are interpreted differently depending on the test‐takers racial or ethnic group—have been more commonly debated in the US context, particularly in response to systemic inequalities in education and healthcare. However, such approaches are not standard practice in many commonly used paediatric cognitive assessments across Europe. Nonetheless, ensuring representativeness in normative samples and cultural fairness in assessment practices remains a key consideration across all contexts.

Additionally, a greater emphasis should be placed on enhancing accessibility, particularly in low‐resource settings, by developing scalable, culturally appropriate assessment tools that minimise bias related to early exposure to literacy, formal education, and cultural background. Existing tools, such as the European Cross‐Cultural Neuropsychological Test Battery (CNTB), the Multicultural Neuropsychological Scale (MUNS), and the BENCI battery, provide a foundation for such efforts [12, 13, 14]. However, similar systematic efforts are lacking for paediatric populations. For example, tools such as the Raven's Coloured Progressive Matrices Test, though used across various cross‐cultural settings with young people, still exhibit cultural bias that impacts test performance [15]. While nonverbal tools currently in use offer options for assessing children from diverse linguistic backgrounds, there remains a significant gap in the systematic adaptation and validation of paediatric neuropsychological assessments across cultural contexts. Many existing tools are not fully standardised for cross‐cultural use, and even nonverbal measures may still carry cultural assumptions or be influenced by prior educational exposure. Future research should focus on adapting and validating culturally responsive child‐specific neuropsychological assessments to ensure equitable and accurate cognitive evaluations across diverse backgrounds.

## 2 Developmental Considerations in Cross‐Cultural Contexts

Cognitive development is influenced by both biological and environmental factors, with cultural influences playing a key role in cognitive domains such as memory, attention, problem‐solving, and language acquisition [16, 17]. However, most neuropsychological assessments are predominately normed within specific cultural and educational systems, which raises concerns about their generalisability across diverse populations [18]. Research on the adaptation of neuropsychological testing, including the WISC‐V, highlights that variations in educational experiences and cognitive strategies across cultures can lead to differences in test performance, underscoring the need for culturally responsive assessment methods [5, 19, 20]. Literacy rates and educational experiences differ within and between cultures [21], impacting cognitive development and neuropsychological test performance [19]. Children raised in environments with limited early exposure to written language—due to differences in home learning environments, migration background, or educational access—may show different cognitive profiles than peers with more consistent engagement in literacy‐based activities. In this context, literacy reflects broader variation in early educational opportunities rather than formal reading ability alone. These differences suggest that assessments should account for diverse developmental trajectories rather than relying solely on benchmarks derived from a single cultural context.

Research suggests that cognitive development follows distinct trajectories depending on educational systems, parental expectations, and cultural practices [22, 23, 24]. For instance, assessments relying on analogical reasoning or verbal fluency may disadvantage children from cultures with strong oral narrative traditions, where knowledge transmission prioritises spoken narratives over written texts. Similarly, problem‐solving tasks assuming familiarity with hierarchical classification systems—such as taxonomic categorisation rather than functional grouping—may not align with the cognitive strategies reinforced in all cultures. Additionally, early educational experiences shape executive functioning and other cognitive abilities, with structured schooling environments supporting the development of different developmental trajectories than those where formal early education is less emphasised [25, 26, 27]. These findings highlight the importance of establishing culturally appropriate developmental norms to ensure accurate assessment of cognitive abilities across diverse contexts.

Additionally, upon searching the existing literature, there appears to be a paucity of empirical studies examining the impact of cultural heritage on children's neuropsychological test performance [2]. This gap in research highlights the need for clinicians to:

- *Examine the Relevance of Normative Data:* Many assessments rely on developmental norms that may not reflect the educational and linguistic experiences of children from different cultural backgrounds. Clinicians should be aware of whether available norms are representative of the population they are assessing and exercise caution when interpreting results from assessments standardised in different contexts.
- *Assess Literacy and Educational Backgrounds:* Since literacy and educational quality vary widely, clinicians should consider alternative methods for evaluating cognitive skills in children with limited formal education or different literacy experiences. This may involve using nonverbal tasks or culturally adapted assessments. By integrating nonverbal and culturally adapted assessment tools with a thorough developmental, educational, and socio‐cultural history, psychologists can more accurately distinguish between the impact of formal education and cognitive difficulties.
- *Recognise Culturally Specific Cognitive Strategies:* Some problem‐solving approaches, memory strategies, and categorisation methods differ across cultures. Assessments should accommodate these differences rather than assume universal cognitive patterns where possible. For example, Indigenous Australian children often use spatial memory strategies based on oral navigation traditions, which may not align with assessments prioritising verbal and sequential recall [28].
- *Minimise Language and Cultural Bias in Testing:* Assessments should be adapted to reduce reliance on language‐heavy tasks where verbal ability or familiarity with specific cultural knowledge could disadvantage the child. Clinicians should consider supplementary measures, such as nonverbal problem‐solving tasks or culturally adapted verbal tests, to obtain a more accurate picture of cognitive abilities. However, caution is still necessary, as bias has been observed even in so‐called culture‐free tests administered to young people [15]. In addition to adapted and nonverbal assessments, qualitative and observational methods can provide useful complementary data. Direct observations of behaviour during assessment, input from parents or carers and teachers, and culturally informed clinical interviews can help contextualise test results. For example, recording a child's problem‐solving strategies, engagement, and persistence during tasks may reveal cognitive strengths not captured through scoring alone. Integrating such information supports a more holistic understanding of a young person's functioning, particularly when normative data are limited or test performance may be confounded by language or cultural unfamiliarity.

Additionally, to improve paediatric neuropsychological assessments for diverse populations, research should focus on:

- *Developing Culturally Responsive Norms:* Large‐scale studies should establish normative data sets that account for variations in education, linguistic background, and cultural practices, ensuring assessments reflect typical development across different populations. Without representative data, assessments risk misclassifying typical developmental variation as impairment, especially in children from underrepresented groups. This can contribute to diagnostic inequity and misallocation of resources. Normative samples must reflect the diversity of the populations clinicians serve, considering not just ethnicity but intersecting factors such as language exposure, socioeconomic status, and migration background.
- *Investigating Socio‐Cultural Influences on Development:* Further research is needed to understand how cultural and environmental factors shape cognitive development, allowing for a more accurate interpretation of assessment results.
- *Better Defining and Measuring Bilingualism and/or Multilingualism:* Understanding a child's language ability is fundamental to accurate neuropsychological assessment. Language ability is multidimensional, encompassing expressive and receptive skills, fluency, vocabulary, grammar, and pragmatic use, and influenced by age of acquisition, exposure, and language dominance across contexts [29]. A comprehensive assessment should integrate validated proxy‐report measures, such as parent or teacher questionnaires assessing language exposure, use, and acculturation, alongside objective language tasks. In older children and adolescents, validated self‐report measures may also be appropriate. Future research should focus on refining these protocols to ensure a more accurate interpretation of bilingual and multilingual children's cognitive performance.
- *Creating More Inclusive and Adaptable Assessment Tools:* Future assessments should incorporate tasks that minimise bias related to language, literacy, and cultural familiarity. This could involve modifying existing tools by replacing culturally specific items with universally recognisable stimuli (e.g., adapting picture naming tests), designing new nonverbal measures like visual pattern recognition or spatial reasoning tasks, and incorporating dynamic testing approaches that assess learning potential rather than relying solely on static test scores. Dynamic assessment approaches, which focus on learning processes rather than fixed performance, are particularly useful in cross‐cultural contexts. These methods involve a test‐teach‐retest format to evaluate a child's capacity to learn when provided with instruction, helping to distinguish between a lack of prior exposure and genuine cognitive difficulty. This is especially relevant in cases where children have had limited formal education, are bilingual or multilingual, or come from non‐Western educational traditions. Dynamic assessment offers a culturally responsive alternative to static testing, promoting equity in cognitive evaluation by focusing on developmental potential rather than existing knowledge shaped by cultural experience.

By integrating these considerations into clinical practice and research, neuropsychological testing with young people can become more equitable, reducing the risk of misdiagnosis and ensuring that children receive assessments and interventions that are appropriate for their developmental and cultural contexts.

## 3 Interpreter‐Mediated Assessment in Paediatric Neuropsychology

A recent European Consortium on Cross‐Cultural Neuropsychology (ECCroN) paper has highlighted the complexities of interpreter‐mediated neuropsychological assessments [30]. Unlike adults, children's language proficiency varies significantly with age and educational exposure, making standardised testing through interpreters more complex. Young children may have difficulty understanding test instructions when interpreters use indirect translations or paraphrase the original wording. This may result in inconsistent responses and potentially compromise the validity of the assessment. Guidelines for interpreter‐mediated assessments must be adapted to include strategies for using developmentally appropriate language, training for interpreters to understand typical and atypical child development, and structured methods to maintain test standardisation.

Building on the paper by Nielsen and colleagues [30], we have considered additional factors specific to paediatric neuropsychology in interpreter‐mediated assessments:

- *Language Development in Children:* Unlike adults, children's language abilities develop with age and schooling. Interpreters need to use language suited to the child's developmental level and avoid paraphrasing test instructions, which can affect test accuracy.
- *Cultural Differences in Test Behaviour:* Children's responses to interpreters and testing environments may be influenced by cultural expectations around communication and authority. Pre‐assessment briefings can help ensure interpreters use appropriate phrasing and consider cultural factors that may affect performance.
- *Determining the Need for an Interpreter:* Clinicians need structured methods to assess when a child requires an interpreter or an adapted tool, as conversational fluency does not always indicate proficiency in the language required for cognitive testing. This includes considering the child's language dominance, input from parents and teachers, and acculturation level.
- *Interpreter Training in Child Assessment:* Interpreters working in paediatric neuropsychology should have an understanding of child development and neuropsychological testing to ensure accurate translation of instructions and responses while maintaining standardised administration. While interpreter training in child development and neuropsychological assessment is ideal, it may not be feasible in all contexts due to variability in interpreter education and resources. Developing scalable training packages, including short modules focused on child communication and test standardisation principles, may help address this gap while acknowledging potential resource constraints.
- *Reducing the Risk of Misdiagnosis:* Without appropriate adaptations, language‐related performance differences may be mistaken for cognitive impairment. Implementing structured approaches, such as using validated language proficiency assessments, gathering information from parents and teachers about the child's language use in different contexts, and ensuring pre‐assessment briefings with interpreters to align on test administration procedures, can help prevent inaccurate assessments. Additionally, ensuring interpreter competency through specialised training in neuropsychological testing and child development is essential to maintaining test validity and reliability.

These additional considerations highlight the need for paediatric‐specific interpreter protocols to improve the accuracy, reliability, and cultural fairness of neuropsychological assessments in children. The forthcoming British Psychological Society (BPS) guidelines on working with interpreters, which will include neuropsychological assessments for the first time, mark a significant step forward in addressing gaps in culturally appropriate neuropsychological practice [31]. These guidelines will be particularly relevant for paediatric assessments, where interpreter‐mediated testing presents unique challenges. Internationally, standards such as the American Educational Research Association Standards for Educational and Psychological Testing have also addressed interpreter use. Additionally, some local European organisations may provide national guidance on interpreter use. However, the availability of such guidance and its specificity to neuropsychological assessment vary. Clinicians and researchers outside the UK may wish to consult their country's regulatory body or adapt the BPS recommendations to fit local regulatory and clinical frameworks.

It is also important to consider that interpreter‐mediated assessments may not always be the most reliable approach for diagnosing developmental conditions in children from diverse linguistic backgrounds. For example, in the case of suspected developmental language disorder in bilingual children, some evidence suggests that direct parental interviews may provide more accurate diagnostic information than direct testing with interpreters [32]. This highlights the need for flexible, case‐sensitive approaches that draw on multiple information sources, including parental reports, teacher input and culturally informed developmental histories.

## Author Contributions

**Freddie O'Donald:** conceptualization, methodology, formal analysis, writing – original draft, investigation, visualization, validation. **Clara Calia:** investigation, writing – review and editing, validation. **Sanne Franzen:** writing – review and editing, visualization. All authors have read and approved the final version of the manuscript.

## Conflicts of Interest

The authors declare no conflicts of interest. All authors are members of the European Consortium on Cross‐Cultural Neuropsychology (ECCroN), which aims to improve the assessment of culturally, educationally, and linguistically diverse individuals across Europe.

## Transparency Statement

The lead author Freddie O'Donald, Clara Calia affirms that this manuscript is an honest, accurate, and transparent account of the study being reported; that no important aspects of the study have been omitted; and that any discrepancies from the study as planned (and, if relevant, registered) have been explained.

## Data Availability

Data sharing is not applicable to this article, as no new data were created or analyzed in this study.
